# Genome-wide association and RNA-seq analyses reveal a potential gene related to linolenic acid in soybean seeds

**DOI:** 10.7717/peerj.16138

**Published:** 2023-11-02

**Authors:** Di Qin, Jiehua Xing, Ping Cheng, Guohui Yu

**Affiliations:** 1 Innovative Institute for Plant Health, Zhongkai University of Agriculture and Engineering, Guangzhou, Gongdong, China; 2 Guangdong Provincial Key Laboratory of Plant Adaptation and Molecular Design, Guangzhou University, Guangzhou, Guangdong, China

**Keywords:** Soybean, Linolenic acid, GWAS, RNA-seq, MALDI-TOF IMS, Fatty acid biosynthesis

## Abstract

Linolenic acid (LA) has poor oxidative stability since it is a polyunsaturated fatty acid. Soybean oil has a high LA content and thus has poor oxidative stability. To identify candidate genes that affect the linolenic acid (LA) content in soybean seeds, a genome-wide association study (GWAS) was performed with 1,060 soybean cultivars collected in China between 2019–2021 and which LA content was measured using matrix-assisted laser desorption/ionization time-of-flight imaging mass spectrometry (MALDI-TOF IMS). A candidate gene, *GmWRI14*, encoding an APETALA2 (AP2)-type transcription factor, was detected by GWAS in cultivars from all three study years. Multiple sequence alignments showed that *GmWRI14* belongs to the plant WRI1 family. The fatty acid contents of different soybean lines were evaluated in transgenic lines with a copy of *GmWRI14*, control lines without *GmWRI14*, and the *gmwri14* mutant. MALDI-TOF IMS revealed that *GmWRI14* transgenic soybeans had a lower LA content with a significant effect on seed size and shape, whereas *gmwri14* mutants had a higher LA content. compared to control. The RNA-seq results showed that *GmWRI14* suppresses *GmFAD3s* (*GmFAD3B* and *GmFAD3C*) and *GmbZIP54* expression in soybean seeds, leading to decreased LA content. Based on the RNA-seq data, yeast one-hybrid (Y1H) and qRT-PCR were performed to confirm the transcriptional regulation of *FAD3s* by GmWRI14. Our results suggest that *FAD3* is indirectly regulated by *GmWRI14*, representing a new molecular mechanism of fatty acid biosynthesis, in which *GmWRI14* regulates LA content in soybean seeds.

## Introduction

Soybeans (*Glycine max* (L.) Merrill) are one of the most important oil crops grown worldwide, particularly in China, and soybean oil now accounts for more than 50% of the total vegetable oil consumption (Liu et al., 2020; Zhao et al., 2019). Approximately 5–11% of soybean oil contains linolenic acid (LA) (Zhang et al., 2018; Clemente & Cahoon, 2009). LA is an essential polyunsaturated fatty acid that acts at multiple levels to alter membrane fluidity and cause inflammation (De Groot et al., 2004; Chan, Bruce & McDonald, 1991). Soybean oil with a high LA concentration degrades easily at high temperatures during the frying cycle, causing a rancid odor (Tompkins, Maskan & Karataş, 1998; Yang et al., 2009; Marangoni et al., 2020). The n-3 double bonds in LA facilitate its combination with oxygen molecules, resulting in poor oxidative stability (Thomsen et al., 1999; Do et al., 2019). Decreasing LA content is important for soybean seed aging and for improving the oxidative stability of soybean oil (Singh et al., 2001). Therefore, investigating the genetic basis of LA content in soybeans has become an important goal for breeding soybeans with a low LA content that are resistant to high temperatures (Sivaraman et al., 2004). Some potential candidate genes associated with LA content in soybeans have been identified in previous genome-wide association analyses (GWAS) (Singh et al., 2001; Li et al., 2019), such as fatty acid desaturase-3 genes (*FAD3*). Previous studies showed that ω3-fatty acid desaturase increases LA levels (Lakhssassi et al., 2017; Wen et al., 2018; Yang et al., 2018). In addition, many transcription factors (TFs) regulate *FAD3* expression and affect LA accumulation, such as bZIP transcription factor *bZIP67*, *ABA INSENSITIVE3 (ABI3)* and *leafy cotyledon1* (*LEC1*). These genes are highly correlated with LA synthesis and may affect *FAD3* expression or may indirectly influence linolenic acid content by cooperating with other TFs that regulate fatty acid synthesis. An important example is *WRINKLED1* (*WRI1*), which is correlated with fatty acid content and lipid synthesis regulation in different plant seeds (Kong, Yuan & Ma, 2019; Kong et al., 2020). Previous studies have also found that LA levels were higher in *wri1* mutant seeds, but with lower total oil volume (Tang et al., 2019; Kuczynski et al., 2020; Guo et al., 2020). However, these studies do not completely explain the function of *WRI1*, and additional potential target genes and cooperative TFs need to be explored. For instance, fatty acid desaturase (*FAD2*), Leafy Cotyledon1 (*LEC1*) and stearoy-acyl-carrier-protein desaturase (*SAD*) expression have closely related to *WRI1*, potentially implicating *WRI1* in the regulation of different fatty acids in soybean seeds (Zhang et al., 2019; Kong & Ma, 2018; Chen et al., 2018).

In our study, we used GWAS to screen candidate genes associated with LA content in soybeans from 2019–2021 to, and discovered a new candidate gene, the APETALA2 (AP2)-type transcription factor *GmWRI14*. RNA-seq and qRT-PCR results indicated that *GmWRI14* suppressed the expression of both *GmFAD3s* and *bZIP54* in soybean seeds, with the inhibition of *GmFAD3s* leading to lower LA content. This study not only lays a theoretical foundation for improving the seed oil quality of soybeans but also provides effective strategies and genetic resources to break the mutual constraints between oil content and quality.

## Materials and Methods

### Determining fatty acid content of different soybean cultivars

A total of 1,060 soybean lines from China were collected to determine their fatty acid content. To obtain more accurate phenotypic data for the GWAS, soybean lines were planted in three regions of China representing typical climates (subtropical, tropical, and temperate zones). The seeds were planted at the following locations: 2019–2020 on 124.65°E, 43.14°N; in 2020–2021 on 114.03°E, 38.29°N; in 2021–2022 on 113.01°E, 24.17°N. After harvesting, the seeds of different soybean lines were dried and the fatty acid content was measured. Five fatty acids (linolenic acid, stearic acid, oleic acid, palmitic acid, and linoleic acid) in soybean cultivar seeds were determined using matrix-assisted laser desorption/ionization time-of-flight imaging mass spectrometry (MALDI-TOF IMS). The correlation coefficients of fatty acids were calculated using SPSS software (SPSS Inc., Chicago, IL, USA). Each calculation was repeated thrice to ensure accuracy.

### Genotyping and GWAS analysis

We used the CTAB method to extract genomic DNA from fresh leaves of each soybean line (Porebski, Bailey & Baum, 1997). The Beijing Biomarker Biotechnology Co. Ltd. (Beijing, China) provided the sequencing services. A total of 2,935,226 SNP markers with a minor allele frequency (MAF) > 0.06, obtained from genotyping, were screened for GWAS using the fastlmmc model. GWAS was used to analyze the association between the genotype and phenotype datasets. The Haploview 4.2 software was used to construct a Manhattan map. The threshold value was set to *–log(p)* > 4.20. The plink2 software was used to calculate the LD decay distances. The NR database and Swiss-Prot were used to search for candidate genes in the 650 kb genomic region of the important SNPs.

### Plant transformation

The *GmWRI14* gene from the soybean cultivar 010a (approval number 2012010) was cloned into the *BamHI-SacI* site of plasmid pTF101 named *pTF101- GmWRI14-Flag*, which was induced by the *CaMV35S* promoter, and the target gene was terminated by the *NOS* terminator. The recombinant plasmid was transformed into calli using *Agrobacterium tumefaciens* strain LBA4404 (Holsters et al., 1978). Individual T_0_ plant lines were established in a greenhouse, and three independent transgenic soybean lines with the same plasmid were harvested: *GmWRI14-1, GmWRI14-*2, and *GmWRI14-3*.

### The generation and identification of *GmWRI14* gene-edited lines

Following a previous protocol, the novel CRISPR/Cas9 Vector pGES201 was used for soybean genome editing (Do et al., 2019). CRISPRdirect (http://crispr.dbcls.jp) was used to design the target site sequence based on the exon sequence of the *GmWRI14* gene in the soybean genome. The *GmWRI14* gene from soybean cultivar 010a (approval number 2012010) was cloned into the *BamHI-SacI* site of plasmid pTF101 named *pTF101-GmWRI14-3×Flag*, which was induced by the *pM4* promoter, and the target gene was terminated by the *NOS* terminator. The CRISPR/Cas9 expression vector was constructed using the method described by Bai et al. (2020). The CRISPR/Cas9 expression vector was transformed into *Agrobacterium tumefaciens* LBA4404 using Agrobacterium-mediated soybean cotyledon node genetic transformation method (Holsters et al., 1978). The EasyPure® Plant Genome DNA Kit (TransGen, Beijing, China) was used according to the manufacturer’s instructions to extract total genomic DNA from the seed samples of the putative mutants. The T_0_–T_2_ generation seeds and control seeds of the transgenic plants were planted in a greenhouse under a 16/8-h light/darkness cycle. When the T_1_–T_2_ generation plants grew the first triple-compound leaf, they were screened for glyphosate resistance. The genomic DNA of genetically modified soybeans and controls was extracted using Kangwei Century Company’s new plant genome DNA extraction kit, and PCR amplification was performed using DNA as a template to detect positive plants. Positive plants were selected and the PCR products were sent to a sequencing company for sequencing. DSDecodeM was used to analyze the sequences of the T3 generation plants to characterize CRISPR/Cas9-induced mutations. Successfully edited strains were identified using sequence peaks and were aligned to the reference sequence. Heterozygous mutants exhibited overlapping peaks near the target site, whereas homozygous mutants exhibited a single peak at the target site. Three independent *gmwri14* soybean mutant lines containing the same plasmid were harvested. They are *gmwri14-1*, *gmwri14-2*, and *gmwri14-3*.

### The RNA-seq data analysis

To analyze the transduction pathways induced by *GmWRI14*, the total RNA of each sample (*GmWRI14* transgenic soybeans, CK, and *gmwri14* mutant) was isolated from the seeds for RNA sequencing, with three biological and three technical replicates, using the Super total RNA® extraction kit (TaKaRa, San Jose, CA, USA). A total of 2 μg RNA was obtained from each plant sample for the RNA sequencing. RNA quality was assessed using Nanodrop 2000c (Thermo Fisher Scientific, Waltham, MA, USA). All transcriptome data were analyzed and calculated using methods outlined in previous studies (Zhong et al., 2011; Sultan et al., 2012; Kumar et al., 2012). The RNA-sequencing depth was 10×, and the 2^−ΔΔCt^ method was used for the fold change of gene expression (Livak & Schmittgen, 2001). The original data were deposited in the China National GenBank database (accession number PRJNA435633). Each sample had an average of 5.68 GB of data for a total of 102.11 GB of sequencing data. We used bioinformatics software (https://www.bioinformatics.babraham.ac.uk/projects/trim_galore/) to improve the data quality, resulting in 41.66 GB of clean data. The clean data of all samples reached 6 GB, and the base percentage of Q30 was more than 93%. The statistical power of this experimental design was calculated in cluster Profiler Power with a *q value* $\leq$ 0.01. A KEGG statistical analysis (Kyoto Encyclopedia of Genes and Genome) and Gene Ontology analysis (http://www.geneontology.org/) were performed to identify the functions of the differentially expressed genes (DEGs).

### Gene expression analysis by qRT-PCR

After 25 days of seedling growth, RNA samples were extracted from the seeds of soybean lines with low LA content and high LA content using an RNA extraction kit (TaKaRa, San Jose, CA, USA). Using overexpressed *GmWRI14* soybeans, *gmwri14* mutants, and control soybean seeds as materials, total RNA was extracted and reverse transcribed into cDNA at 10, 11, 12, 13, and 14 days after podding, using 24-h sampling (T0, T4, T8, T12, T16, T20, and T24 soybean seeds, respectively). qRT-PCR primers for *bZIP54*, *GmFAD3C* and *GmFAD3B*, were used to perform a 24-h expression regulation analysis of the candidate target genes of GmWRI14. qRT-PCR was performed with three biological replicates, according to the MIQE guidelines (Bustin et al., 2009). Real-time fluorescence analysis was performed using a LightCycler (Roche, Basel, Switzerland). The *lectin* gene (Gene ID: 100775957) was used as an internal control (Qin et al., 2022). All primers used are listed in the additional files (Table S1).

### Yeast one-hybrid (Y1H) assay

To test whether GmWRI14 could affect the expression of *FAD3s* by binding to the promoter region of *FAD3s*, both the *FAD3*s (*GmFAD3B* and *GmFAD3C*) and the *GmbZIP54* promoter regions were amplified from soybean cultivar 010a (Approval number 2012010). The Y1H assay was performed according to the manufacturer’s instructions (Zhao et al., 2019).

## Results

### Identification of candidate gene *GmWRI14* by GWAS

**Figure 1:**
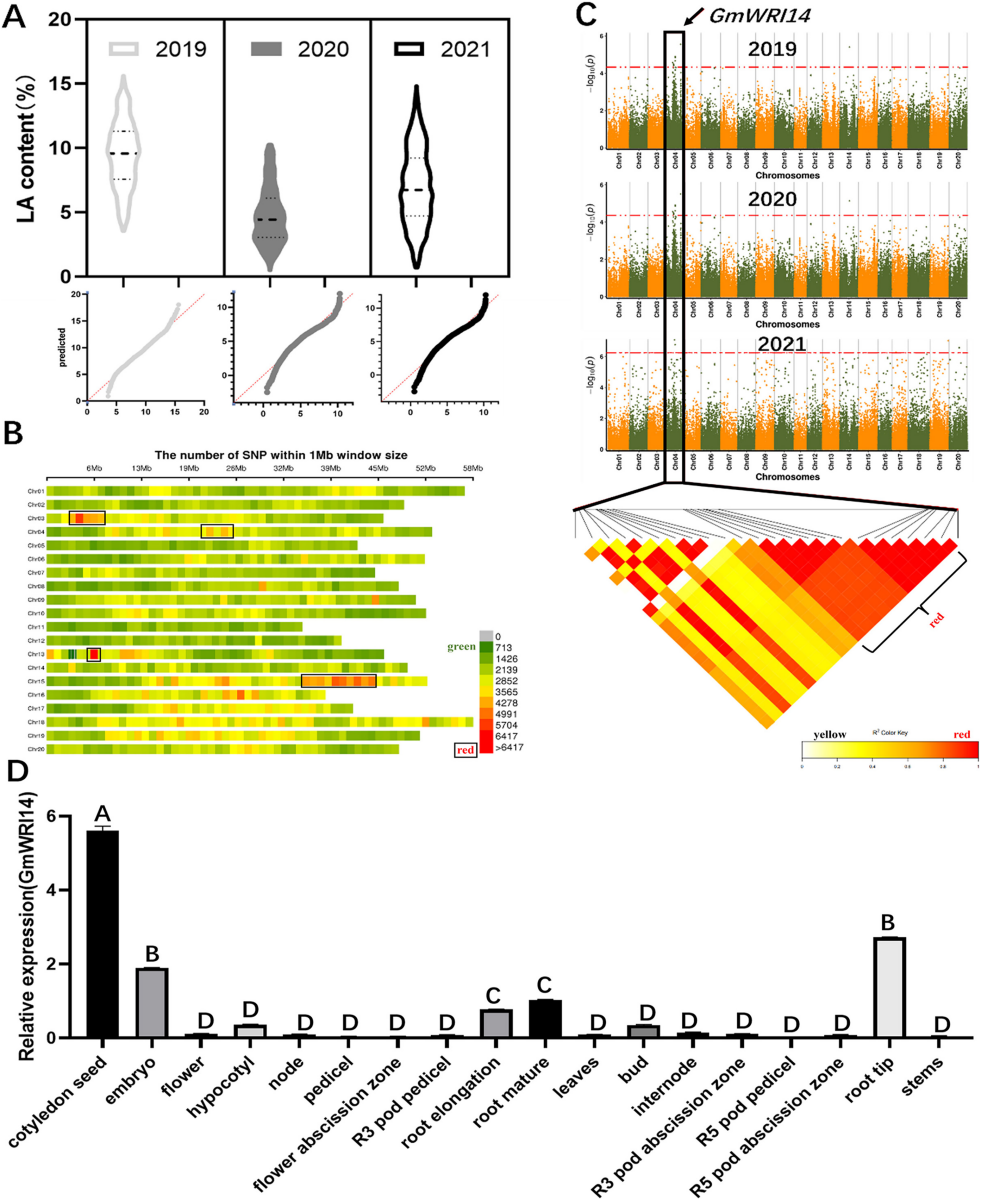
Linolenic acid (LA) content and GWAS analysis from soybean germplasms collected from 2019–2021. (A) The floating range of LA content of 1,060 soybean germplasms collected from 2019–2021. LA content conforms to normal distribution. In 2019, LA content ranged from 2.62–15.75%; in 2020, LA content ranged from 0.25–10.32%; and in 2021, LA ranged from 0.31–16.12%. (B) Density distribution map of SNP on 20 chromosomes of soybean. The green area indicates that the number of SNPs is less than 1,426. (C) GWAS was used to screen candidate genes related to LA from 2019–2021, using the fastlmmc model. Each point represents an SNP on each chromosome. The threshold line is the P-value considered for significance. (D) Analysis of expression of *GmWRI14* in different tissues. *GmWRI14* was mainly expressed in soybean seeds, with an expression of 5.8 ± 0.2. The uppercase letters in (D) represent significant differences (*P* < *0.01*).

The LA content in 1,060 soybean lines collected from different regions of China between 2019 and 2021 ranged from 1.01% to 15.92%. These 3-year measurements suggest that there is a wide variation in LA content in the soybean population, the distribution is continuous, and the LA content conforms to a normal distribution (Fig. 1A). A total of 2,923,211 SNP markers were obtained from the dataset (Fig. 1B), of which 899,218 SNPs had an MAF > 6%. To identify potential candidate genes associated with LA, a GWAS was used to screen candidate genes using the fastlmmc model. These SNP markers were evaluated for their association with LA content. We identified 17 candidate genes associated with LA content from to 2019–2020, ten candidate genes from to 2020–2021, and ten candidate genes from to 2021–2022. Ten SNPs were associated with LA, all of which were located on chromosome 4. *Glyma.04G127300.1*, a candidate gene for LA, was detected by GWAS in each of the 3 years (Fig. 1C), indicating that *Glyma.04G127300.1* is a candidate gene for LA. The results of the bioinformatics analysis showed that *Glyma.04G127300.1* belongs to the plant WRI1 family containing two AP2/EREB domains; therefore, we named it *GmWRI14*. The expression of the *GmWRI14* gene in the flowers, internodes, stems, and roots of the soybean plants was investigated using qRT-PCR. *GmWRI14* was expressed in different organs of soybean plants but was mainly expressed in soybean seeds, with low levels of *GmWRI14* expression detected in the other organs (Fig. 1D). The qRT-PCR results showed that the *GmWRI14* gene expression was highly tissue-specific in soybeans. The raw qRT-PCR data are available in File S1.

**Figure 2:**
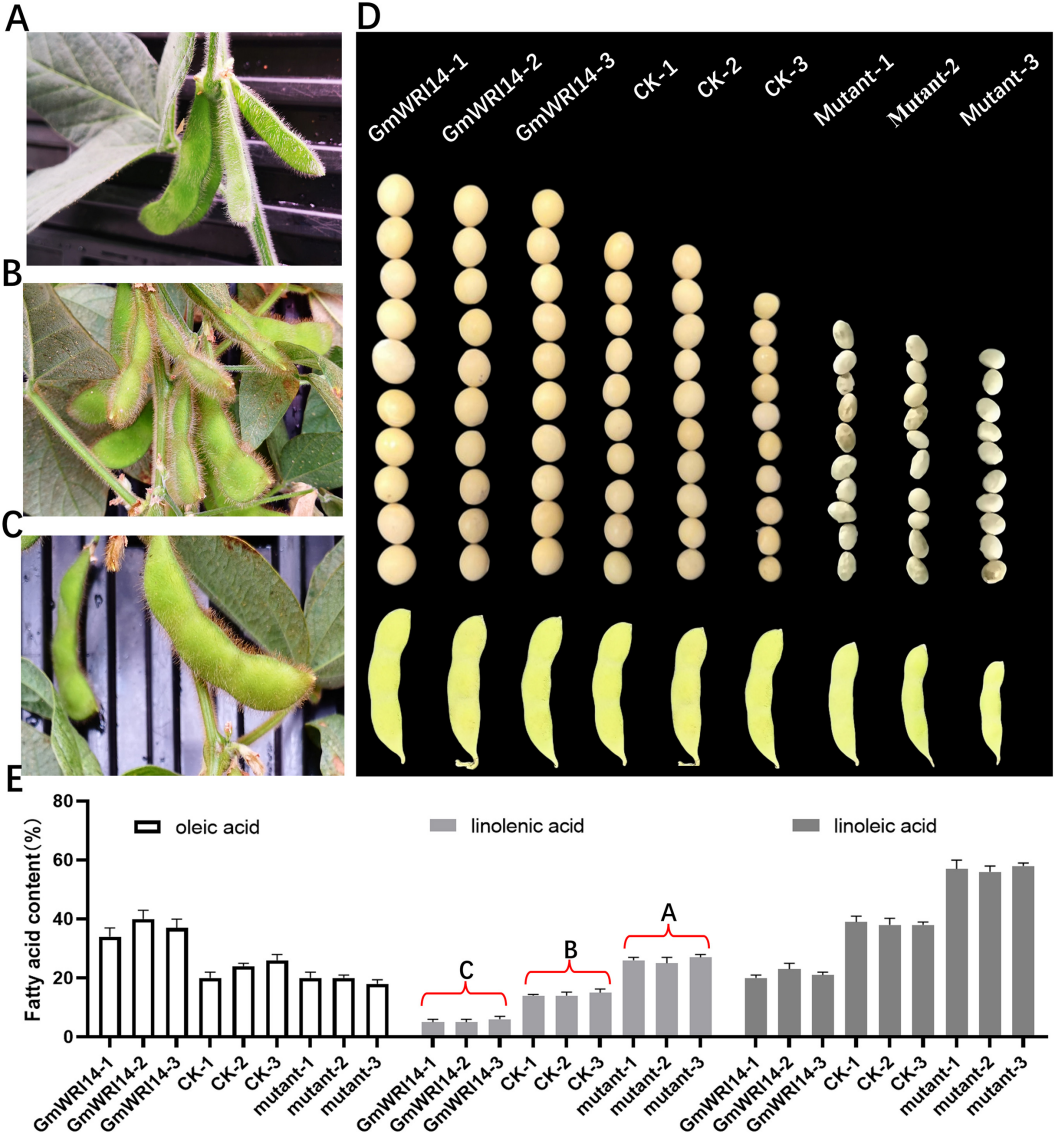
Phenotyping of CK, *GmWRI14* transgenic lines, and *gmwri14* mutant. (A) Phenotype of *gmwri14* mutant. (B) Phenotype of CK. (C) Phenotype of *GmWRI14* transgenic soybean plants. (D) Phenotype of soybean seed from three varieties (CK, *GmWRI14* transgenic lines, *gmwri14* mutant). (E) LA content of different soybean lines. *GmWRI14-1, GmWRI14-2* and *GmWRI14-3* are three independent transgenic soybean lines with the same plasmid. *gmwri14-1 mutant, gmwri14-2 mutant* and *gmwri14-3 mutant* are three independent transgenic soybean lines with the same CRISPR/Cas9 expression vector. The uppercase letters in (E) represent significant differences (*P* < *0.01*).

The *GmWRI14* gene from the soybean cultivar 010a (Approval number 2012010) was cloned into the *BamHI-SacI* site of the plasmid pTF101, named *pTF101- GmWRI14-Flag* (Fig. S1). *GmWRI14* transgenic soybean and *gmwri14* mutant were then produced (Figs. 2A–2D), and Southern blotting was used to detect *GmWRI14* expression (Fig. S2), and MALDI-TOF MS was used to analyze the LA distribution in soybean seeds (Fig. S3). MALDI-TOF IMS results showed that the total fatty acid content of *GmWRI14* transgenic soybean increased from 41.32% to 56.02% (Fig. 2E); however, the LA content of *GmWRI14* transgenic soybean decreased from 12.24% to 6.12%. MALDI-TOF IMS results showed that, compared to soybean receptors (CK), the linolenic acid content in the *gmwri14* mutant significantly increased from 21.01% to 23.92% (Fig. 2E). These results indicate that the expression of *GmWRI14* and its ability to regulate specific genes are important for determining LA content in soybean seeds.

### Overexpression of *GmWRI14* suppresses *GmFAD3s* and *bZIP54* expression in soybean seeds

**Figure 3:**
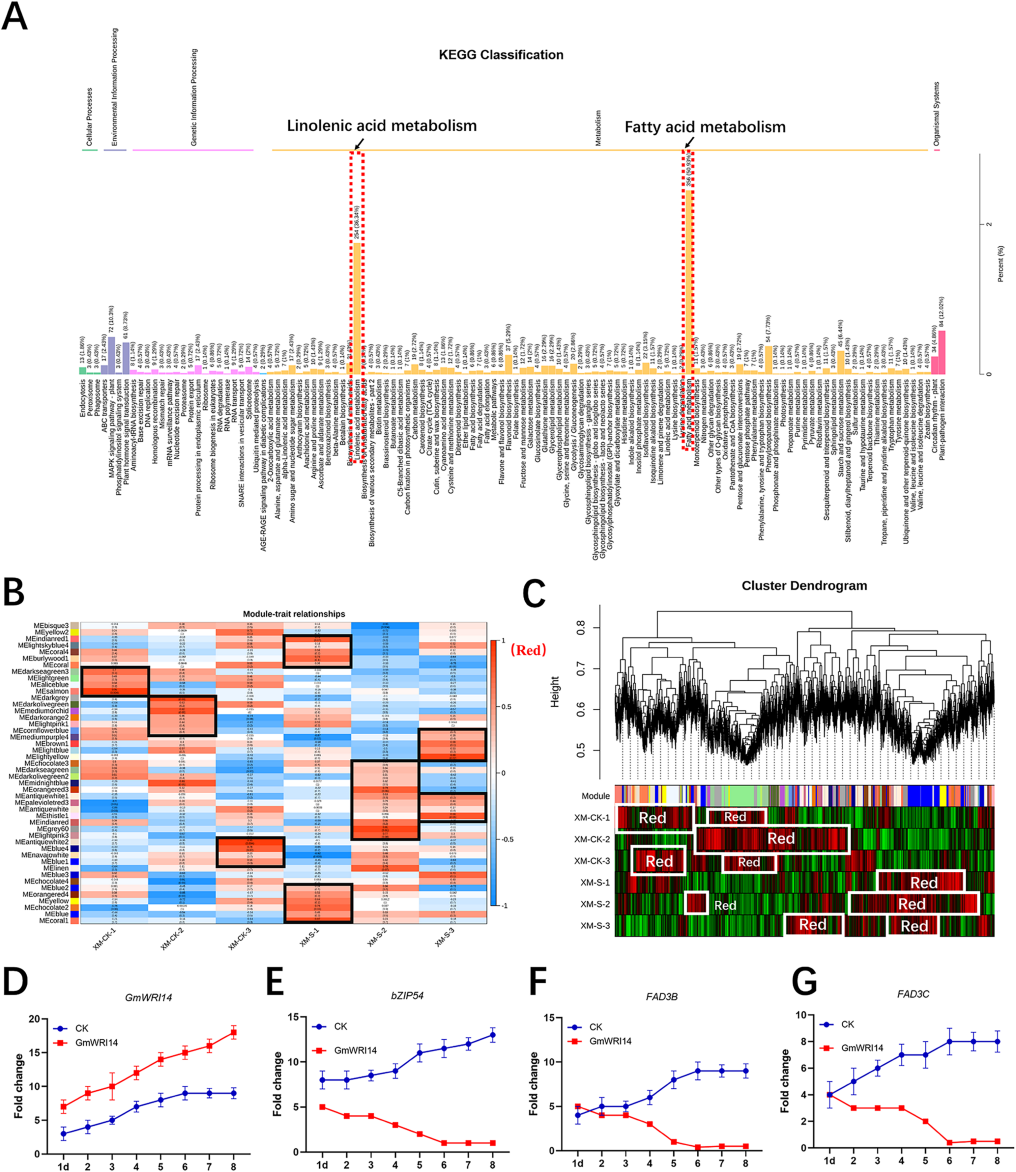
KEGG enrichment analysis and weighted gene co-expression network analysis (WGCNA). (A) The KEGG enrichment analysis shows the differentially-expressed genes are mainly concentrated in fatty acid metabolism and linolenic acid metabolism (red area). (B) The module-sample correlation heatmap. The color scale on the right shows correlations from −1 (red) to 1 (blue). (C) Dendrogram of co-expression modules identified by WGCNA. (D–G) the expression of *GmFAD3B, GmFAD3C* and *GmbZIP54* genes in *GmWRI14* transgenic soybean. 1d = 1 day.

RNA-seq was used to verify the genes related to fatty acid synthesis that were induced by *GmWRI14* in transgenic soybean seeds and control plants (CK, *GmWRI14* transgenic lines, and *gmwri14* mutant). RNA extracted from the seeds was used for RNA-seq to analyze the differentially expressed genes (DEGs) between the three varieties (CK, *GmWRI14* transgenic lines, and *gmwri14* mutant). The RNA-seq results showed that the expression of more than 50 genes related to fatty acid synthesis changed significantly. The KEGG and GO analyses revealed that the 891 identified DEGs were mainly involved in fatty acid and linolenic acid metabolism (Fig. 3A). Weighted correlation network analysis (WGCNA) was performed to identify *GmWRI14* co-expressed genes using the WGCNA R package. The correlation coefficient between any two genes was calculated using WGCNA to analyze the expression patterns of different genes. The square of the threshold of the correlation coefficient was 0.8, and 15,134 potential genes were transferred to the co-expression network to explore the relationship between phenotypes and candidate genes. A gene co-expression correlation matrix was defined by calculating the dissimilarity coefficients of different nodes, and a hierarchical clustering tree was constructed. Abnormal samples were removed based on clustering tree results. Different colors were used to distinguish the 13,211 genes, with gray representing the genes that could not be classified into any module (Fig. 3B). The 13,211 genes were divided into 45 distinct modules (Fig. 3B), and the turquoise, yellow, and light-yellow modules had the highest correlations with LA content in the module–trait correlation analysis (Fig. 3C). RNA-seq showed that DEGs related to linolenic acid metabolism were differentially expressed (more than 2.5 times), namely *GmFAD3C* and *GmFAD3B* (Fig. S4). qRT-PCR showed that the expression of *GmWRI14* was significantly increased in the seeds of *GmWRI14* transgenic soybean lines (Fig. 3D), and the expression of *GmbZIP54* in *GmWRI14* transgenic soybean seeds on different days was also measured by qRT-PCR (Fig. 3E), with *GmbZIP54* expression ranging from 2.45 ± 0.52 to 8.44 ± 0.22. The expression of *GmFAD3C* and *GmFAD3B* was significantly decreased in the seeds of *GmWRI14* transgenic soybean lines (Table S2), with *GmFAD3C* expression ranging from 22.75 ± 0.26 to 26.12 ± 0.26 and *GmFAD3B* expression ranging from 4.12 ± 0.15 to 9.23 ± 0.13 in *GmWRI14* transgenic seeds (Figs. 3F and 3G). The expression levels of *GmFAD3B* and *GmFAD3C* were 24% and 31% higher, respectively, in control seeds than in the *GmWRI14* transgenic soybean lines (Table S2). Notably, *GmFAD3B* and *GmFAD3C* expression remained virtually unchanged in the stems and leaves of *GmWRI14* transgenic soybeans, with *GmFAD3B* and *GmFAD3C* expression in seeds ranging from 11.25 ± 0.41 29.22 ± 0.25. qRT-PCR results suggested that the *GmWRI14* gene inhibited the expression of *GmFAD3s* (*GmFAD3C* and *GmFAD3B*) in soybean seeds (Table S2).

### Disruption of *GmWRI14* altered total fatty acid content, and expression of *GmFAD3s* increased

**Figure 4:**
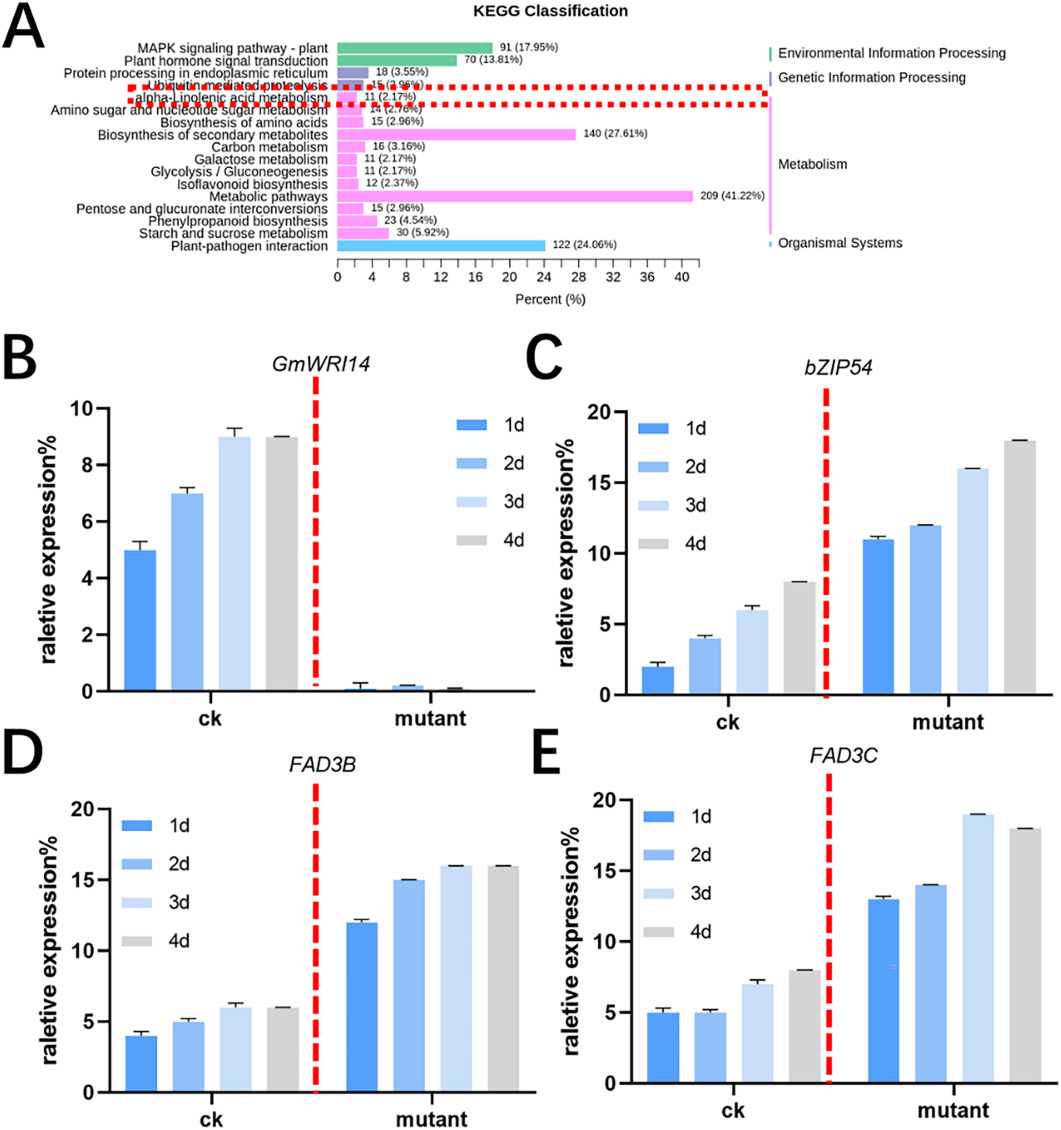
KEGG classification in *gmwri14* mutant soybean plants and qRT-PCR for DEGs. (A) The functional characterization of *GmWRI14* mutant soybean for a KEGG enrichment analysis. (B) The relative expression of *GmWRI14* in *gmwri14* mutant soybean seeds. (C) The relative expression of *GmbZIP54* in *GmWRI14* mutant soybean plants. (D) The relative expression of *GmFAD3B* in *gmwri14* mutant soybean plants. (E) The relative expression of *GmFAD3C* in *gmwri14* mutant soybean plants.

MALDI-TOF MS was used to profile fatty acid species in the mature seeds of *gmwri114* mutant and control soybean lines. There was a significant difference between the *gmwri114* and wild-type soybeans (*P* < *0.01*). The LA content increased from 11.31% to 20.59% in the *gmwri14* mutant, and owing to the reduction in total fatty acids, the size and quality of the *gmwri14* mutant seeds were also reduced. The RNA-seq data showed that in *gmwri14* mutant soybean seeds, the DEGs related to fatty acid synthesis from the two varieties (CK and *GmWRI14* mutant) changed significantly (Fig. 4A), with a threshold of >2.5-fold change indicating significance. In the *gmwri14* mutant, qRT-PCR results indicated that the lack of *GmWRI14* induced the transcriptional activity of *GmFAD3s* or *GmbZIP54* genes (Figs. 4B–4D). *GmWRI14* is a transcriptional inhibitor that negatively regulates the expression of *FAD3s*. The abundance of *FAD3s* genes is closely related to linolenic acid content in different plant seeds (Flores et al., 2008; Hu et al., 2006). Therefore, the *GmWRI14* gene likely regulates linolenic acid synthesis by regulating *FAD3s* genes. The qRT-PCR results suggested that disruption of *GmWRI14* enhanced the expression of *GmFAD3s* and *GmbZIP54* in soybean seeds.

### GmWRI14 can inhibit *GmFAD3s* in the presence of *GmbZIP54*

**Figure 5:**
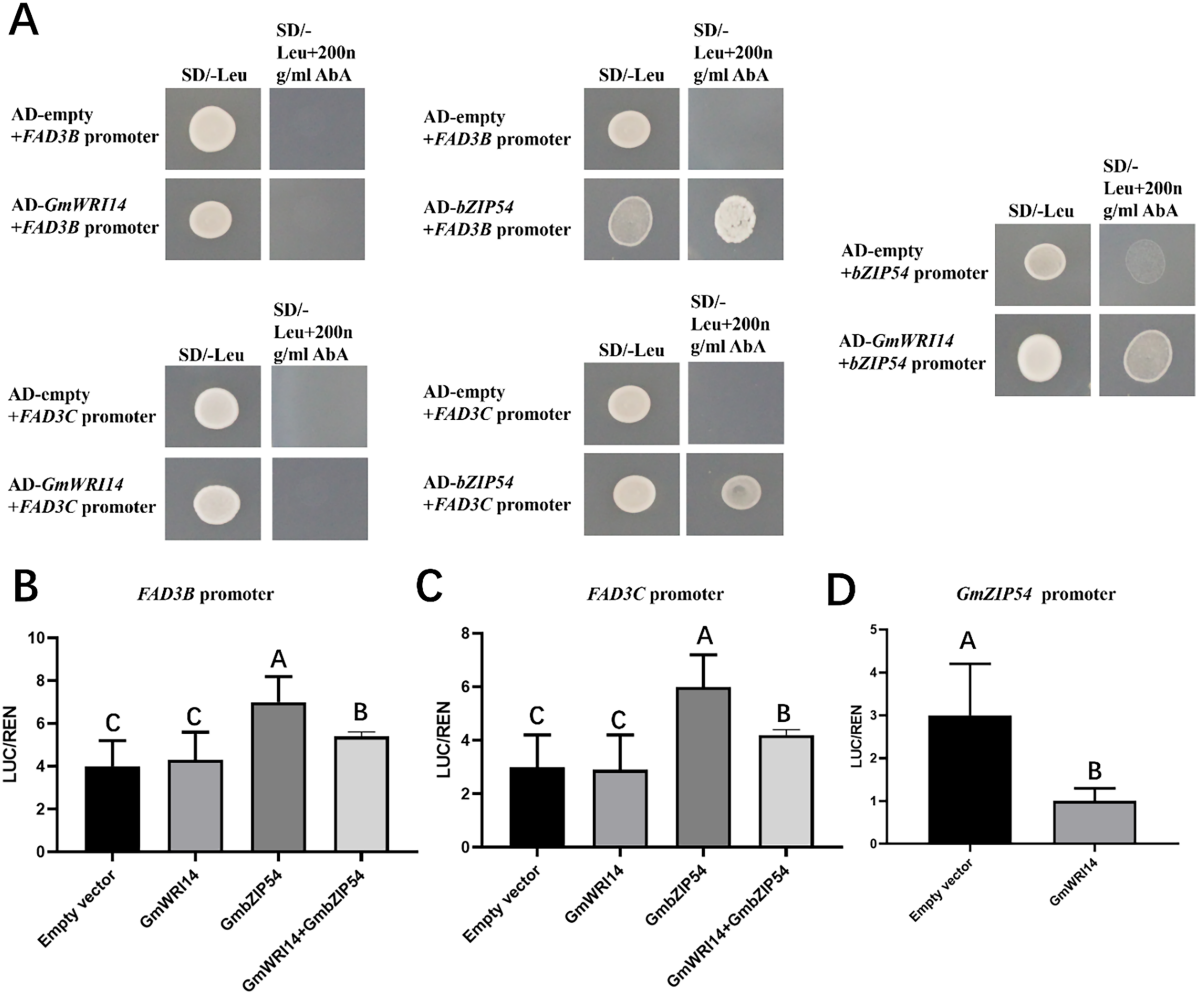
*GmWRI14* inhibited *GmFAD3s* (*GmFAD3B* and *GmFAD3C*) in the presence of GmbZIP54. (A) The yeast one-hybrid assay indicates there is no interaction between GmWRI14 and *GmFAD3s* (*GmFAD3B* and *GmFAD3C*) promoter. It also indicates an interaction between GmWRI14 and the *GmbZIP54* promoter, and between *GmbZIP54* and *GmFAD3s* (*GmFAD3B* and *GmFAD3C*) promoter. (B and C) Transient dual-luciferase detections of *GmFAD3B, GmFAD3C*, and *GmbZIP54* promoters in *Nicotiana benthamiana* leaves. The relative LUC/REN activity was lower than that of the empty vector, indicating that GmbZIP54 directly binds to the *GmFAD3s* (*GmFAD3B* and *GmFAD3C*) promoter region as a transcription activator of GmbZIP54. (D) The relative LUC/REN activity was lower than that of the empty vector, indicating that GmWRI14 directly binds to the GmbZIP54 promoter region as a transcription inhibitor of GmWRI14. Values are means ± SDs, *n* = 3. The uppercase letters in (B)–(D) represent significant differences (*P* < *0.01*).

To investigate whether the *GmWRI14* gene binds to the promoter regions of *GmFAD3B*, *GmFAD3C*, and *GmbZIP54* genes in soybean, a yeast one-hybrid assay (Y1H) was performed (Fig. 5A). The results showed that the *GmWRI14* gene activated the *GmbZIP54* promoter region, but not the promoter regions of the *GmFAD3s* genes. *GmbZIP54* directly bound to the promoter region of *GmFAD3s* genes (Fig. 5A). When *GmFAD3s* were co-infiltrated with *GmbZIP54*, the promoter regions of the *GmFAD3s (GmFAD3B* and *GmFAD3C)* were significantly activated (Figs. 5B and 5C), with the promoter regions of the *GmbZIP54* were significantly suppressed. The raw data for the dual-luciferase assay are shown in Table S3. Together, these data suggest that *GmWRI14* inhibits the expression of *GmFAD3s* genes by interacting with *GmbZIP54*, leading to decreased linolenic acid production, and that strong inhibition of *FAD3s* can potentially be achieved by the combined action of *GmbZIP54* and *GmWRI14*.

## Discussion

An estimated 62–77% of the fatty acid content in soybean seeds is polyunsaturated, with linoleic acid comprising 50–58% and linolenic acid comprising 10–12% of the fatty acid content. Linolenic acid (LA, 18:3) is an important component of soybean seeds. It is an essential fatty acid necessary for both oil quality and seed aging (Clemente & Cahoon, 2009). LA also results in a low oil oxidation stability (Tompkins, Maskan & Karataş, 1998). Breeders have screened several genes associated with LA using GWAS (Zeng et al., 2017; Wang et al., 2020; Yu et al., 2019; Zhang et al., 2019). Soybean oil with a low linolenic acid content has great market potential because it is more stable and resistant to high temperatures (Hammond et al., 2018; Perkins, 2000). *FAD3* encodes the key enzymes involved in linolenic acid synthesis in different plants, and *FAD3* expression levels directly affect linolenic acid and linoleic acid content in seeds (Thapa et al., 2018). During fatty acid metabolism in plants, *FAD3* genes are under the control of some TFs such as *FUS3* and *ABI3*, and the cooperation of different types of TFs plays a key role in LA anabolism.

The A9PETALA2 (AP2)-type transcription factor WRINKLED1 (WRI1) was the first positive regulator of fatty acid biosynthesis in plants (Chen et al., 2018; Cerna & Benning, 2004; An & Suh, 2015; Baud et al., 2009). The high sequence similarity observed between the AP2 DNA-binding domains of WRI1, WRI2, WRI3, and WRI4 suggests that these four factors may recognize and bind to similar cis-regulatory elements (To et al., 2012). However, WRI1 is the only member of the WRI clade that triggers high rates of acyl chain production in seeds (To et al., 2012). A previous study found that WRI1 was markedly induced in developing seeds, whereas WRI2, WRI3, and WRI4 mRNAs was barely detectable. Another study showed that WRI1 is the only member of the WRI family that participates in the activation of fatty acid biosynthesis in maturing embryos, providing acyl chains for triacylglycerol (TAG) production (To et al., 2012). WRI1 can also bind to the AW box to activate several target genes involved in fatty acid synthesis at the onset of seed maturation. The *WRI1* gene can both directly regulate the synthesis rate of fatty acids and works with a variety of transcription factors to indirectly regulate the synthesis and metabolism of fatty acids (Baud et al., 2009). Many target genes upregulated or downregulated by WRI1 have been discovered by RNA-seq and qRT-PCR, such as *pPK, TAL, E1, E2, BS, MCMT, SAD*, BLISTER (*BLI*) and Arabidopsis hemoglobin 1 (*AtGLB1*) (Ye et al., 2020; To et al., 2012).

Compared with the promotional effect of *WRI1*, the inhibitory effect of *WR1* has not been extensively studied. More attention is usually paid to the impact of *WRI* on the total oil content of plants, rather than its possible function in regulating the biosynthesis of different fatty acids, or whether the regulation of individual fatty acids explains *WRI*’s impact of WRI on total oil content. In this study, we used GWAS and RNA-seq to identify potential candidate genes associated with LA content in soybean seeds. We identified a previously unidentified candidate gene, *GmWRI14*, that is related to LA content. The amino acid sequence encoded by *GmWRI14* was compared to *AtWRI1* (Gene ID:824599). High sequence similarity was observed between the AP2 DNA-binding domains of *AtWRI1* and GmWRI14 (homology of the amino acid sequences between *GmWRI14* and *AtWRI1* was 62.34%). suggested that *GmWRI14* may recognize and bind to a similar cis-regulatory element, leading to a significant increase in seed oil content. In addition, *WrI1* seeds have an altered fatty acid composition, with higher levels of linolenic acid (C18:3) and erucic acid (C22:1), but lower levels of oleic acid (C18:1) and linoleic acid (C18:2) (Deng et al., 2019), indicating that the relationship between *WRI1* and fatty acid metabolism is complex. Compared to the control group, the seeds of *GmWRI14* transgenic soybean lines had a higher total oil content but a 40% lower LA content, resulting in a high oleic acid/linolenic acid ratio. *WRI1* has been shown to be necessary for the regulation of carbon partitioning for fatty acid synthesis in plant seeds (Deng et al., 2019; Chen et al., 2018), but *WRI1* is unable to directly regulate genes associated with fatty acid modification and triacylglycerol (TAG) assembly in the endoplasmic reticulum (Deng et al., 2019), such as Desaturase2 (*FAD2*), *FAD3*, Fatty acid elongase1 (*FAE1*), and Diacylglycerol acyltransferase1 (*Dgat1*), because *WRI1* specifically recognizes the binding cis element AW-box [CnTnG(n)7CG] (Baud et al., 2009). The RNA-seq and qRT-PCR results in this study demonstrated that *GmWRI14* represses *FAD3*, but the promoter region of *FAD3* does not contain an AW-box conserved sequence. Therefore, another transcription factor likely combines with *GmWRI14* to convert it into a repressor. *GmWRI14* may indirectly mediate the regulation of *FAD3s* genes. For example, in *Arabidopsis* seeds, *bZIP* regulates the omega-3 fatty acid content of seed oil by activating *FAD3*.

**Figure 6:**
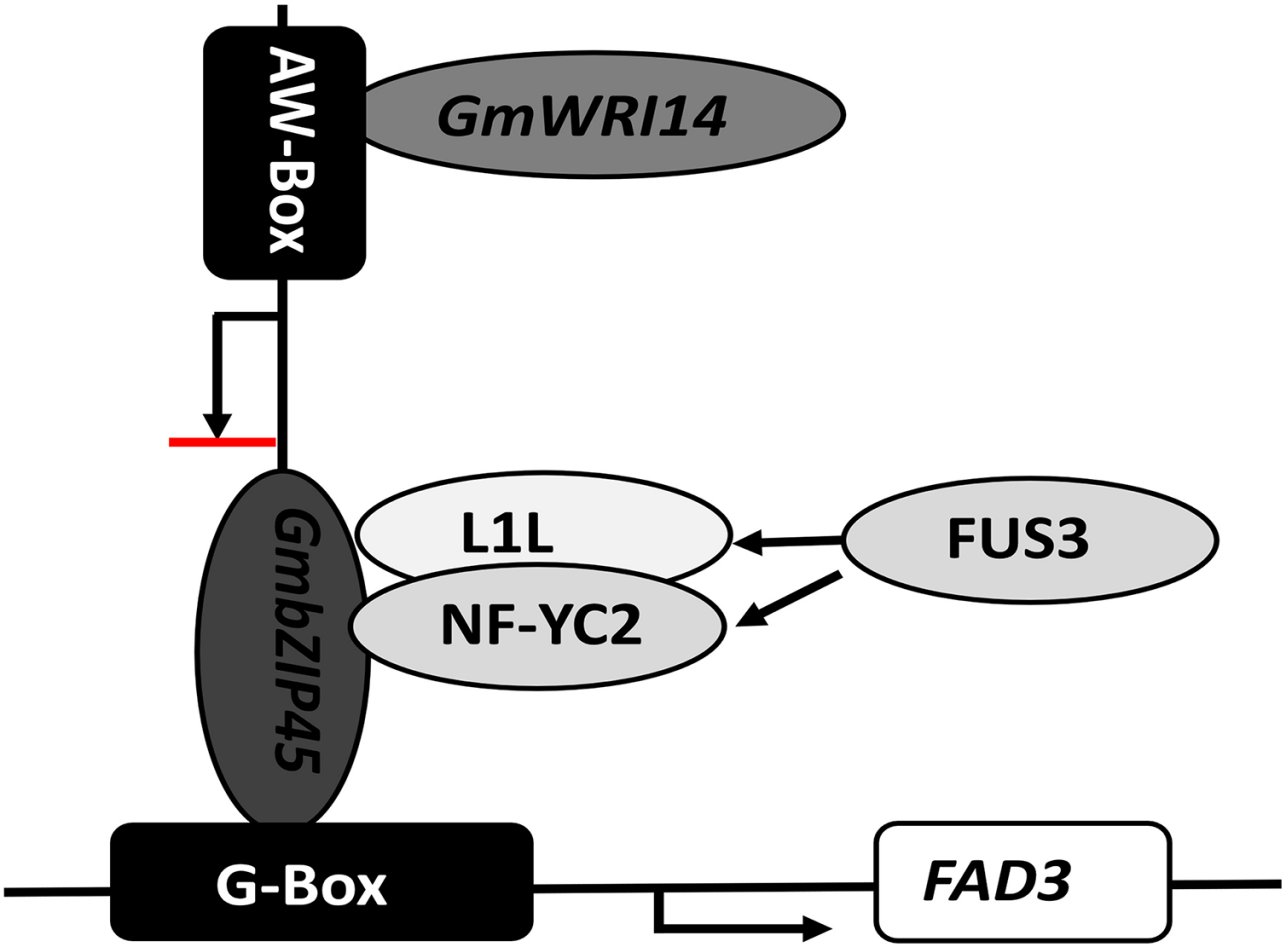
The proposed role of *GmWRI14* in the transcriptional regulation of *FAD3* in soybean seeds. *GmWRI14* could not bind to the *FAD3* promoter region directly, because it lacks an AW-box sequence; *bZIP54* could bind to the *FAD3* promoter region *via* G-Box to improve the *FAD3* expression, and GmWRI14 could bind to the *GmbZIP54* promoter region to inhibit *GmbZIP54* expression, indirectly decreasing the levels of LA production. Here, we show that GmWRI14 binds to the *GmbZIP54* promoter region, influencing the expression of *GmFAD3s*.

In our study, we found that the interaction of *GmbZIP54* with *GmWRI14* resulted in a reduction of *GmbZIP54* activity. Our study also suggests that WRI1 not only improves oil accumulation but also fine-tunes fatty acid biosynthesis by indirectly inhibiting the expression of *FAD3s* in soybean seeds. The LA content of *GmWRI14* transgenic soybean lines was five-fold lower compared of the control group. The qRT-PCR results showed that the expression of *GmFAD3-1B* and *GmFAD3-1C* was significantly reduced in *GmWRI14* transgenic soybeans. When *GmWRI14* was knocked out, there was a significant increase in LA content, and *FAD3* and *bZIP54* expression, which are involved in LA metabolism, was affected. We also showed that *bZIP54* could bind to the *FAD3* promoter region, *GmWRI14* could bind to the AW-box in the *GmbZIP54* promoter region and limit *GmbZIP54* expression, and that *GmWRI14* could inhibit *FAD3* expression in the presence of *bZIP54*. Since *GmWRI14* could not bind to the *FAD3* promoter region because it lacks an AW-box sequence, *bZIP54* could bind to the *FAD3* promoter region to improve *FAD3* expression, and *GmWRI14* could bind to the *bZIP54* promoter region to inhibit *bZIP54* expression, indirectly decreasing the levels of LA production (Fig. 6).

In conclusion, we demonstrated that the *WRI1* gene plays an important role in the structure of fatty acids, which helps to maintain the stability of soybean oil. *GmWRI13* can directly promote the content of seed oil, but also works with other TFs to indirectly regulate the content of specific fatty acids in soybean oil.

## Conclusions

In the present study, a new candidate gene associated with LA content, *GmWRI14*, was identified in soybean seeds using GWAS and RNA-seq. We generated transgenic soybean plants overexpressing *GmWRI14*. These *GmWRI14* transgenic soybeans had a lower LA content. The RNA-seq results indicated that the transcription factor *GmWRI14* inhibited the expression of *FAD3s (GmFAD3B and GmFAD3C)* genes, and that this inhibition is likely the underlying mechanism of WRI1’s contribution to fatty acid homeostasis in soybean oil.

## Supplemental Information

10.7717/peerj.16138/supp-1 Supplemental Information 1 *pTF101- GmWRI14-Flag*. The *GmWRI14* gene from soybean cultivar 010a (Approval number 2012010) was cloned into the *BamHI-SacI* site of plasmid pTF101 named *pTF101-GmWRI14-Flag*, which was induced by the *CaMV35S* promoter, and the target gene was terminated by the *NOS* terminator.

10.7717/peerj.16138/supp-2 Supplemental Information 2 The full-length southern blot was used to detect the GmWRI14 expression. The southern blot was used to detect the *GmWRI14* expression. (A) Southern blot analysis of the copy number of the GmWRI14 expression cassette in T0 plants.(B) Southern blot analysis of the copy number of the GmWRI14 expression cassette in T1 plants.

10.7717/peerj.16138/supp-3 Supplemental Information 3 MALDI-TOF IMS was used to analyze LA distribution in the soybean seeds. The LA content of the *GmWRI14* transgenic soybean decreased compared to soybean receptors (CK), the content of linolenic acid in the *gmwri14* mutant was significantly increased compared to soybean receptors (CK). (A–C) The LA distribution in different soybean lines, (D–F) Ion peaks of LA in MALDI-TOF-MS of different soybean lines.

10.7717/peerj.16138/supp-4 Supplemental Information 4 RNA-seq showed that DEGs related to linolenic acid metabolism were differentially expressed (more than 2.5 times), namely *GmFAD3C* and *GmFAD3B*. (A) RNA-seq data from transgenic soybean of 8 days. (B) RNA-seq data from transgenic soybean of 10 days. Red represents up-regulated, green represents down-regulated.

10.7717/peerj.16138/supp-5 Supplemental Information 5 All primers.

10.7717/peerj.16138/supp-6 Supplemental Information 6 Analysis of gene expression in different days. (A) Analysis of gene expression in different days. (B) Analysis of GmFAD3s and GmbZIP54 expression in different tissues.

10.7717/peerj.16138/supp-7 Supplemental Information 7 Raw data of dual-luciferase.

10.7717/peerj.16138/supp-8 Supplemental Information 8 The GmWRI14 expression in different tissues.

## Additional Information and Declarations

### DNA Deposition

The following information was supplied regarding the deposition of DNA sequences: The group II intron/IEP sequences are available at GenBank: KJ826367 to KJ826395.

### Data Availability

The following information was supplied regarding data availability: The raw measurements are available in the Supplemental Files.
