# Supplementary figures and images for: Genome-wide association and RNA-seq analyses reveal a potential gene related to linolenic acid in soybean seeds
Source: PeerJ. 2023 Nov 2;11:e16138. doi: 10.7717/peerj.16138 (PMC10625760; doi:10.7717/peerj.16138)

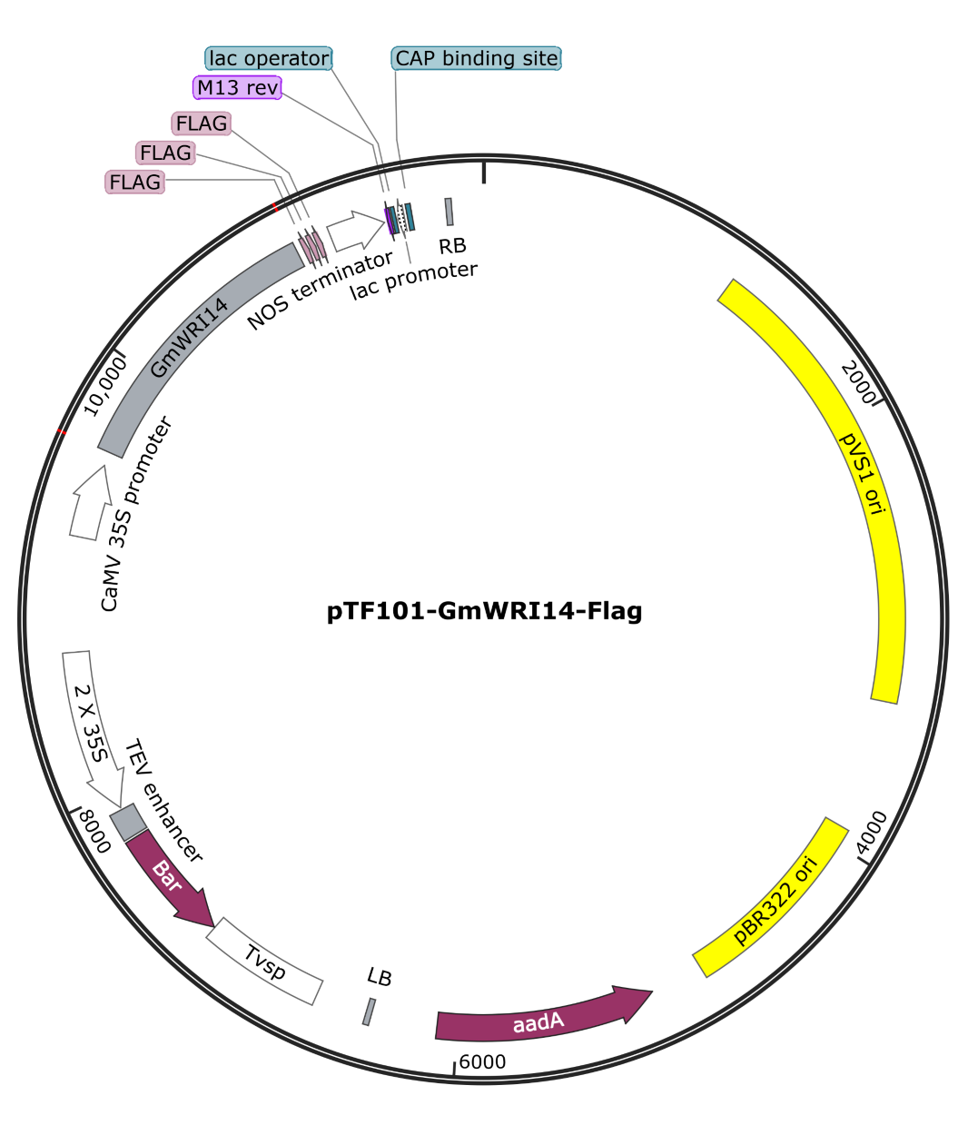

Supplement: Supplemental Information 1 — The GmWRI14 gene from soybean cultivar 010a (Approval number 2012010) was cloned into the BamHI-SacI site of plasmid pTF101 named pTF101-GmWRI14-Flag, which was induced by the CaMV35S promoter, and the target gene was terminated by the NOS terminator. [file peerj-11-16138-s001.png]

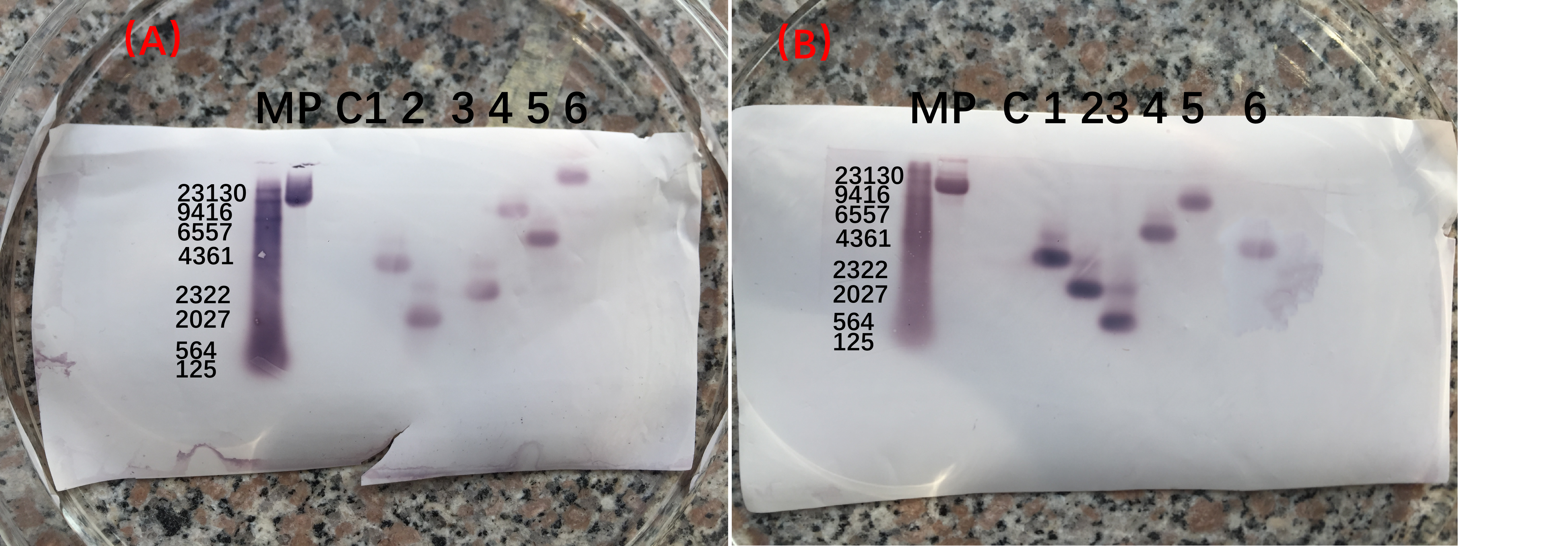

Supplement: Supplemental Information 2 — The southern blot was used to detect the GmWRI14 expression. (A) Southern blot analysis of the copy number of the GmWRI14 expression cassette in T0 plants.(B) Southern blot analysis of the copy number of the GmWRI14 expression cassette in T1 plants. [file peerj-11-16138-s002.png]

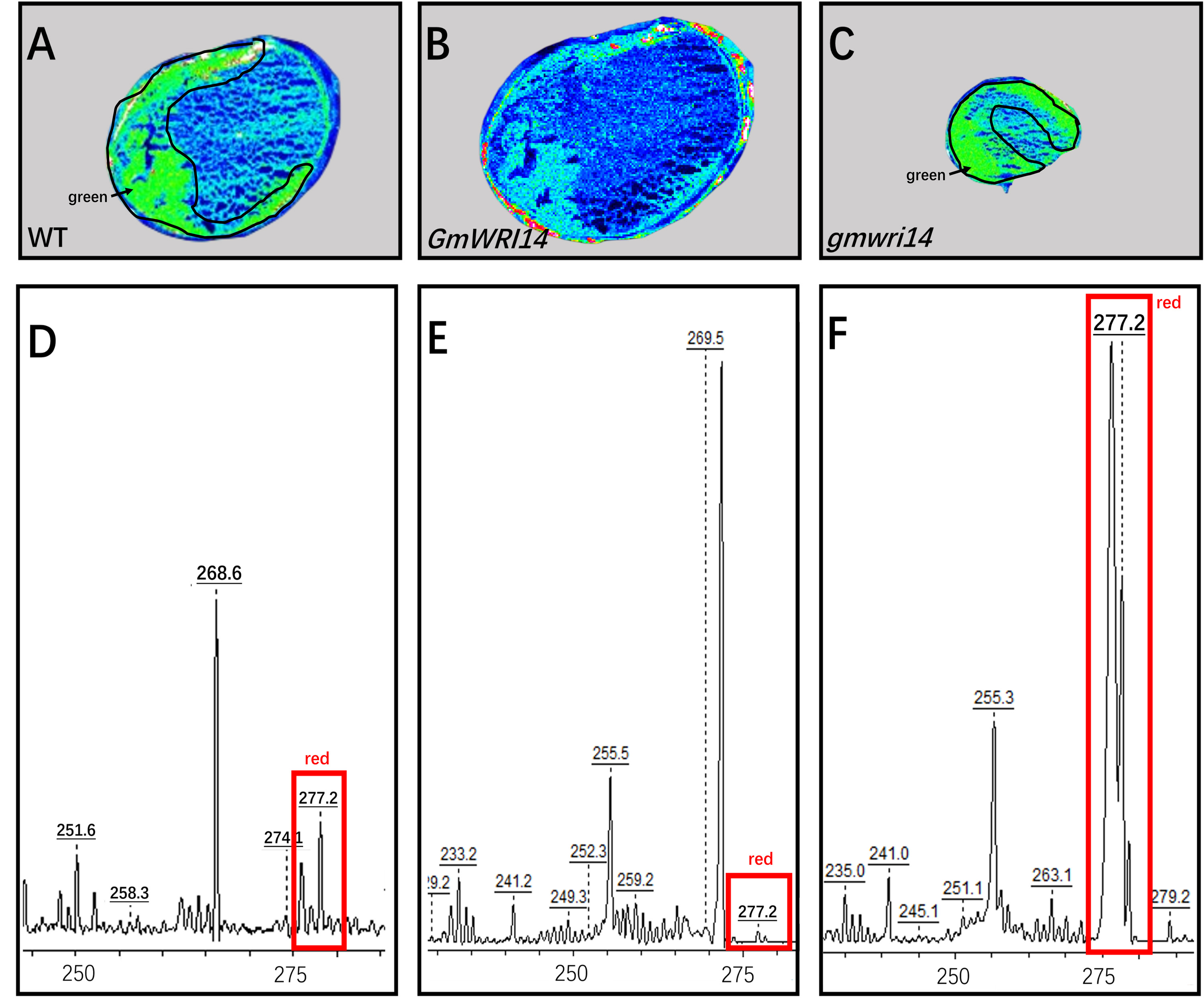

Supplement: Supplemental Information 3 — The LA content of the GmWRI14 transgenic soybean decreased compared to soybean receptors (CK), the content of linolenic acid in the gmwri14 mutant was significantly increased compared to soybean receptors (CK). (A–C) The LA distribution in different soybean lines, (D–F) Ion peaks of LA in MALDI-TOF-MS of different soybean lines. [file peerj-11-16138-s003.jpg]

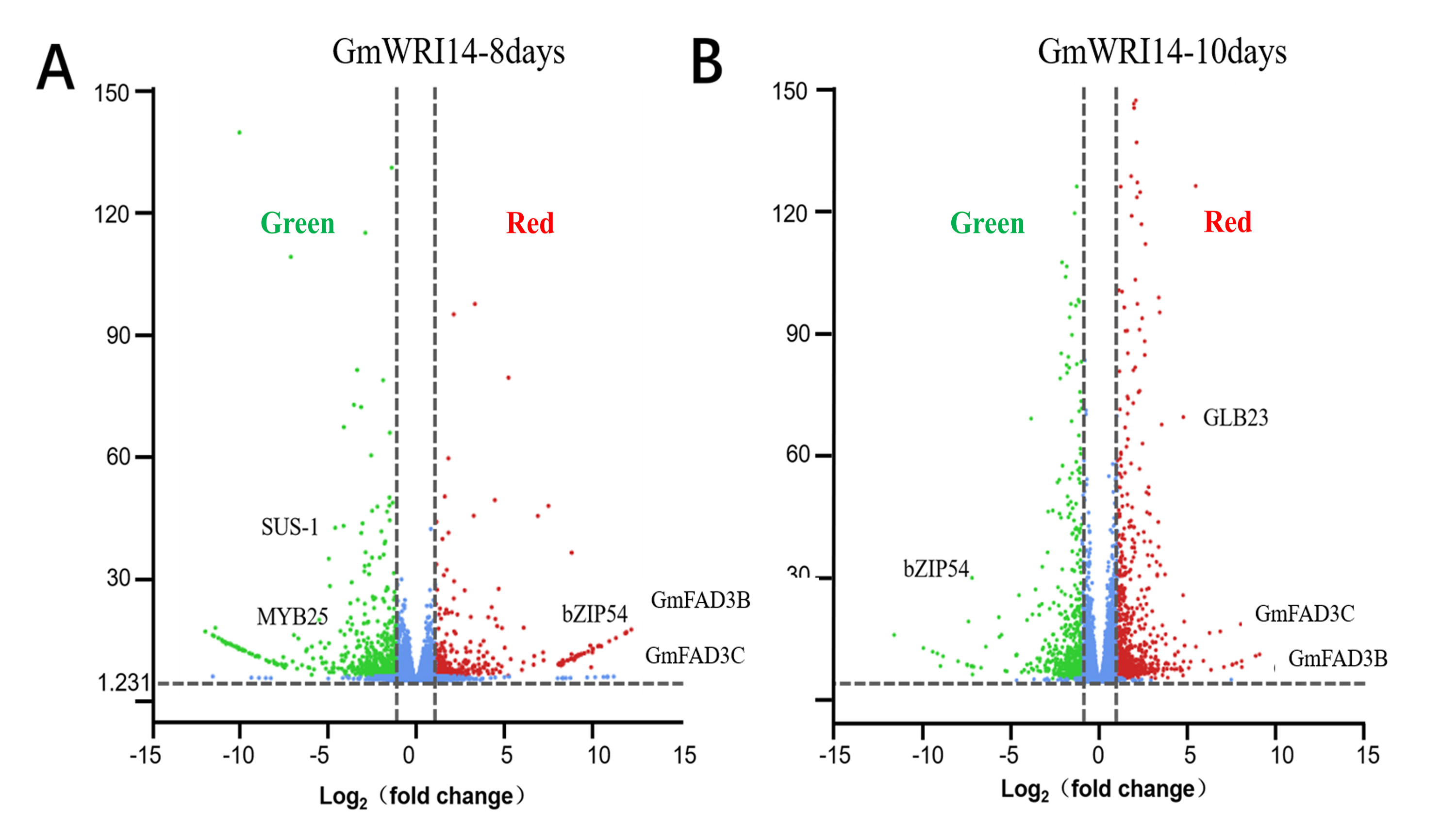

Supplement: Supplemental Information 4 — (A) RNA-seq data from transgenic soybean of 8 days. (B) RNA-seq data from transgenic soybean of 10 days. Red represents up-regulated, green represents down-regulated. [file peerj-11-16138-s004.jpg]
