# Supplementary material for: Genome-wide association and RNA-seq analyses reveal a potential gene related to linolenic acid in soybean seeds
Source: PeerJ. 2023 Nov 2;11:e16138. doi: 10.7717/peerj.16138 (PMC10625760; doi:10.7717/peerj.16138)
Supplement: Supplemental Information 5 [file peerj-11-16138-s005.docx]

| Primer name | Sequence (5^,^-3^,^) |
| --- | --- |
| Lectin- P1 | 5′-GCACTTAAGATACTCTAGGTAC-3′ |
| Lectin- P2 | 5′-CCACCTCCCTACTATCCATT-3′ |
| GmWRI14 -p3 | 5′-TTGCCTGTCTAGATCCACAGCTGGTACCGAT-3′ |
| GmWRI14 -p4 | 5′-TTGTGACCTCGACCTATTGGCGTTACCAATT-3′ |
| GmFAD3B-P5 | 5′-TCCACCTTCATGCTTTGCCGTTACAAGACTCTACT-3′ |
| GmFAD3B-P6 | 5′-TGGATCCGGCTCGCTACCTTCAACTTACTTAACTG-3′ |
| GmFAD3C-P7 | 5′-TATGTATCTCCATTCCTCTTC-3′ |
| GmFAD3C-P8 | 5′-AATCTTCGTTCTGCTGTT-3′ |
| bZIP54-P9 | 5′-CAGGGATCTAAACCTAGTTTCTAGGTAC-3′ |
| bZIP54-P10 | 5′-TCGTTCTGCCCCTACTAAGTACGACATT-3′ |
| WRI14-pM4-T1-F1 | 5′-TGGTCTCGTGCAGATGATGAACAAGAAGATGCGTTTTAGAGCTAGAAATAGC-3′ |
| WRI14-pM4-T1-R1 | 5′-TGGTCTCgcttCAAACCTCCtgcaccagccgggaatcgaa-3′ |
| WRI14-pM4-T2-F1 | 5′-TGGTCTCGAAACTCTTGCTCTGAATGTTGTTCTGCACCAGCCGGGAATCGAA-3′ |
| WRI14-pM4-T2-R1 | 5′-TGGTCTCGGAAGCTCACCTAGTTTTAGAGCTAGAAATAGC-3′ |
| WRI14-pM4-T3-F1 | 5′-TTACGCCAAGCTTGCATGCCGAGGACCTAACAGACTGGCGAAC-3′ |
| WRI14-pM4-T3-R1 | 5′-GTCTTCTGAAaTCGTCGACCTGCAGGGATCTAGTAACATAGATGACACCGCGC-3′ |
| WRI14-pM4-CasF-Test | 5′-GAGGGATTGTAGTTCTGTTG-3′ |
| WRI14-pM4-CasF-Test | 5′-CTATGGCCCATTGGTTGCTC-3′ |

Table S1. All primers for qRT-PCR
