# Supplementary material for: Genome-wide association and RNA-seq analyses reveal a potential gene related to linolenic acid in soybean seeds
Source: PeerJ. 2023 Nov 2;11:e16138. doi: 10.7717/peerj.16138 (PMC10625760; doi:10.7717/peerj.16138)
Supplement: Supplemental Information 6 — (A) Analysis of gene expression in different days. (B) Analysis of GmFAD3s and GmbZIP54 expression in different tissues. [file peerj-11-16138-s006.docx]

| GmWRI14 relative expression | 1d | SD | 2d | SD | 3d | SD | 4d | SD |
| --- | --- | --- | --- | --- | --- | --- | --- | --- |
| ck | 4.3±0.21 | 0.12 | 6.8±0.22 | 0.23 | 8.9±0.22 | 0.56 | 0.88±0.12 | 0.03 |
| mutant | 0.2±0.12 | 0.05 | 0.3±0.23 | 0.02 | 0.6±0.04 | 0.04 | 0.18±0.12 | 0.012 |
|  |  |  |  |  |  |  |  |  |
| bZIP54 relative expression | 1d | SD | 2d | SD | 3d | SD | 4d | SD |
| ck | 2±0.11 | 0.31 | 4±0.13 | 0.22 | 6±0.20 | 0.32 | 8±0.22 | 0.01 |
| mutant | 11±0.21 | 0.52 | 12±0.13 | 0.12 | 16.5±0.22 | 0.11 | 16.3±0.14 | 0.011 |
|  |  |  |  |  |  |  |  |  |
| FAD3B relative expression | 1d | SD | 2d | SD | 3d | SD | 4d | SD |
| ck | 4.2±0.21 | 0.22 | 4.8±0.12 | 0.14 | 6.3±0.14 | 0.13 | 5.8±0.10 | 0.04 |
| mutant | 12.3±0.12 | 0.23 | 14.5±0.10 | 0.02 | 16.1±0.31 | 0.01 | 18±0.12 | 0.001 |
|  |  |  |  |  |  |  |  |  |
| FAD3C relative expression | 1d | SD | 2d | SD | 3d | SD | 4d | SD |
| ck | 4.8±0.23 | 0.23 | 4.7±0.12 | 0.18 | 65.9±0.14 | 0.25 | 6.2±0.10 | 0.11 |
| mutant | 12.2±0.11 | 0.23 | 13.4±0.17 | 0.13 | 17.8±0.21 | 0.41 | 16.2±0.15 | 0.02 |
|  |  |  |  |  |  |  |  |  |

**Table S2-a.** Analysis of gene expression in different days

**Table S2-b.** Analysis of *GmFAD3s* and *GmbZIP54* expression in different tissues

| **Gene** |  | **Soybean Name** | **relative expression in leaves** | | **relative expression in stem** | | |  | **relative expression in roots** | | **relative expression in seed** | | |  | |  |
| --- | --- | --- | --- | --- | --- | --- | --- | --- | --- | --- | --- | --- | --- | --- | --- | --- |
|  | **correlation coefficient with *GmWRI14*** |  | mean | Sig. | mean | Sig | mean | | | Sig. | | mean | Sig. |  |  |  |
| ***GmFAD3B*** |  | Ck | 11.25±0.41 | c | 26.12±0.41 | b | 22.21±0.12 | | | b | | 36.11±0.12 | a |  |  |  |
|  |  | *GmWRI14*-1 | 14.21±0.12 | c | 24.23±0.13 | b | 3.23±1.13 | | | e | | 9.23±0.13 | c |  |  |  |
|  | -0.963--0.991 | *GmWRI14*-2 | 12.14±0.31 | c | 22.21±0.51 | b | 3.23±0.42 | | | d | | 7.32±0.42 | d |  |  |  |
|  |  | *GmWRI14*-3 | 13.14±0.14 | c | 28.23±0.15 | b | 9.11±0.16 | | | d | | 4.12±0.15 | b |  |  |  |
|  |  | *gmwri14*-1 | 13.32±0.11 | c | 24.22±0.16 | b | 26.23±0.23 | | | b | | 34.11±0.16 | a |  |  |  |
|  |  | *gmwri1414*-2 | 12.22±0.12 | c | 28.25±0.31 | b | 24.54±0.26 | | | b | | 35.22±0.61 | a |  |  |  |
|  |  | gmwri14*-3* | 12.35±0.11 | c | 29.22±0.25 | b | 25.51±0.16 | | | b | | 37.23±0.12 | a |  |  |  |
| ***GmFAD3C*** |  | Ck | 12.21±0.34 | c | 27.12±0.12 | b | 23.15±0.64 | | | b | | 28.11±0.32 | b |  |  |  |
|  |  | *GmWRI14*-1 | 16.23±0.21 | c | 23.14±0.17 | b | 11.95±0.54 | | | c | | 22.75±0.26 | b |  |  |  |
|  | -0.893--0.931 | *GmWRI14*-2 | 13.46±0.11 | c | 22.22±0.22 | b | 12.51±0.34 | | | c | | 21.21±0.13 | b |  |  |  |
|  |  | *GmWRI14*-3 | 13.45±0.15 | c | 23.42±0.2 | b | 11.11±0.54 | | | c | | 26.12±0.26 | b |  |  |  |
|  |  | *gmwri14*-1 | 18.14±0.43 | c | 27.27±0.46 | b | 25.34±0.23 | | | b | | 35.12±0.23 | a |  |  |  |
|  |  | *gmwri1414*-2 | 18.66±0.14 | c | 26.66±0.66 | b | 25.56±0.13 | | | b | | 34.55±0.12 | a |  |  |  |
|  |  | *gmwri14-3* | 18.14±0.76 | c | 27.34±0.15 | b | 27.12±0.51 | | | b | | 36.66±0.54 | a |  |  |  |
|  |  | Ck | 0.01±0.02 | f | 0.03±0.04 | f | 0.02±0.01 | | | f | | 8.22±0.02 | d |  |  |  |
|  |  | *GmWRI14*-1 | 0.01±0.15 | f | 0.04±0.02 | f | 0.01±0.01 | | | f | | 2.45±0.52 | e |  |  |  |
|  |  | *GmWRI14*-2 | 0.02±0.01 | f | 0.01±0.02 | f | 0.02±0.01 | | | f | | 3.76±0.32 | e |  |  |  |
| ***GmbZIP54*** |  | *GmWRI14*-3 | 0.01±0.15 | f | 0.04±0.11 | f | 0.01±0.04 | | | f | | 4.44±0.12 | e |  |  |  |
|  |  | *gmwri14*-1 | 0.03±0.02 | f | 0.01±0.02 | f | 0.01±0.01 | | | f | | 16.34±0.22 | c |  |  |  |
|  |  | *gmwri1414*-2 | 0.02±0.11 | f | 0.01±0.15 | f | 0.03±0.05 | | | f | | 12.56±0.31 | c |  |  |  |
|  |  | gmwri14*-3* | 0.01±0.12 | f | 0.03±0.01 | f | 0.02±0.02 | | | f | | 17.76±0.21 | c |  |  |  |

Note: The different lower letters indicate significant differences at *P < 0.05*, as determined by Duncan’s multiple-range test.
