# Supplementary material for: Genome-wide association and RNA-seq analyses reveal a potential gene related to linolenic acid in soybean seeds
Source: PeerJ. 2023 Nov 2;11:e16138. doi: 10.7717/peerj.16138 (PMC10625760; doi:10.7717/peerj.16138)
Supplement: Supplemental Information 7 [file peerj-11-16138-s007.docx]

**Table S3.** Raw data of dual-luciferase

|  | emptey Vector | *GmWRI14* | *GmbZIP54* | *GmWRI14+GmbZIP54* |
| --- | --- | --- | --- | --- |
| FAD3B promoter | 0.0039 | 0.0041 | 0.0063 | 0.0051 |
|  | 0.0051 | 0.0052 | 0.0081 | 0.0052 |
|  | 0.0062 | 0.0054 | 0.0042 | 0.005 |
|  | 0.0036 | 0.0034 | 0.0073 | 0.0048 |
|  | 0.0032 | 0.0036 | 0.0056 | 0.0054 |
|  |  |  |  |  |
|  | emptey Vector | *GmWRI14* | *GmbZIP54* | *GmWRI14+GmbZIP54* |
| FAD3C promoter | 0.0031 | 0.0027 | 0.0069 | 0.0041 |
|  | 0.0032 | 0.0042 | 0.0071 | 0.0042 |
|  | 0.0032 | 0.0034 | 0.0052 | 0.0035 |
|  | 0.0026 | 0.0024 | 0.0053 | 0.0039 |
|  | 0.0032 | 0.0021 | 0.006 | 0.0038 |
|  |  |  |  |  |
|  | emptey Vector | *GmWRI14* |  |  |
|  | 0.0034 | 0.0012 |  |  |
| GmbZIP54 promoter | 0.0042 | 0.0011 |  |  |
|  | 0.0013 | 0.0007 |  |  |
|  | 0.0033 | 0.0011 |  |  |
|  | 0.0038 | 0.0013 |  |  |
